# Arg126 and Asp49 Are Essential for the Catalytic Function of Microsomal Prostaglandin E_2_ Synthase 1 and Ser127 Is Not

**DOI:** 10.1371/journal.pone.0163600

**Published:** 2016-09-29

**Authors:** Joan Raouf, Nazmi Rafique, Michael Christopher Goodman, Helena Idborg, Filip Bergqvist, Richard N. Armstrong, Per-Johan Jakobsson, Ralf Morgenstern, Linda Spahiu

**Affiliations:** 1 Unit of Rheumatology, Department of Medicine Solna, Karolinska Institutet, SE-171 76, Stockholm, Sweden; 2 Unit of Rheumatology, Karolinska University Hospital, SE-171 76, Stockholm, Sweden; 3 Institute of Environmental Medicine, Division of Biochemical Toxicology, Karolinska Institutet, Stockholm, Sweden; 4 Department of Chemistry, Vanderbilt University School of Medicine, Nashville, United States of America; University of Canterbury, NEW ZEALAND

## Abstract

**Introduction:**

Prostaglandins are signaling molecules that regulate different physiological processes, involving allergic and inflammatory responses and cardiovascular control. They are involved in several pathophysiological processes, including inflammation and cancer. The inducible terminal enzyme, microsomal prostaglandin E synthase 1 (MPGES1), catalyses prostaglandin E_2_ production during inflammation. MPGES1 has therefore been intensively studied as a pharmaceutical target and many competitive inhibitors targeting its active site have been developed. However, little is known about its catalytic mechanism.

**Aim:**

The objective of this study was to investigate which amino acids play a key role in the catalytic mechanism of MPGES1.

**Materials and Methods:**

Based on results and predictions from previous structural studies, the amino acid residues Asp49, Arg73, Arg126, and Ser127 were chosen and altered by site-directed mutagenesis. The mutated enzyme variants were cloned and expressed in both the *E*. *coli* and the *Baculovirus* expression systems. Their catalytic significance was evaluated by activity measurements with prostanoid profiling.

**Results and Conclusions:**

Our study shows that Arg126 and Asp49 are absolutely required for the catalytic activity of MPGES1, as when exchanged, the enzyme variants loose activity. Ser127 and Arg73 on the other hand, don't seem to be central to the catalytic mechanism because when exchanged, their variants retain considerable activity. Our finding that the Ser127Ala variant retains activity was surprising since high-resolution structural data supported a role in glutathione activation. The close proximity of Ser127 to the active site is, however, supported since the Ser127Cys variant displays 80% lowered activity.

## 1. Introduction

Prostaglandins (PGs) are signaling molecules that are synthesized *de novo* in mammalian cells from the precursor membrane polyunsaturated fatty acid arachidonic acid (AA) [1] and are important mediators of inflammation. They act as local hormones and regulate different physiological and pathophysiological processes, including allergic [2] and inflammatory responses [3], cardiovascular control [4], as well as platelet aggregation [5]. In the initial step of PG biosynthesis, AA is catalytically converted by cyclooxygenase 1 and 2 (COX-1 and COX-2) to prostaglandin endoperoxide H_2_ (PGH_2_), which is further transformed by the terminal enzyme microsomal prostaglandin E_2_ synthase 1 (MPGES1) to prostaglandin E_2_ (PGE_2_) [6–8]. PGE_2_ is a key mediator of inflammation causing fever, swelling and pain [8–10] and is usually associated with pathophysiological conditions [11].

MPGES1 is a membrane bound enzyme and belongs to the membrane-associated proteins in eicosanoid and glutathione metabolism (MAPEG) superfamily [12]. This superfamily consists of six integral membrane proteins of which five, including MPGES1, use the tripeptide glutathione (GSH) as a substrate or cofactor in their catalytic mechanism. MPGES1 is inducible upon pro-inflammatory stimuli [13] and its expression is highly up-regulated in chronic inflammatory diseases, e.g. rheumatoid arthritis (RA) [13] and cancer [14,15]. For these reasons, MPGES1 has been extensively investigated as a pharmaceutical target. Many competitive inhibitors targeting its active site have been developed [16], though very little is known about its catalytic mechanism.

The enzymatic and mechanistic characterization of MPGES1 is complicated, due to its unstable substrate PGH_2_. Still, understanding the catalytic mechanism of MPGES1 is important for two reasons. The first one is for the potential development of inhibitors that might become future anti-inflammatory drugs and the second, to better characterize and map the catalytic mechanism of MAPEG superfamily members. Several catalytic mechanisms have been proposed for MPGES1. In this study, we have focused on the mechanism proposed by Sjögren *et al*., [17] based on their X-ray crystallography structure at 1.2 Å resolution. These authors suggested that the residues involved in the catalytic activity of MPGES1 would most likely be Asp49, Arg73, Arg126 and Ser127. Inspired by this structure, a novel catalytic mechanism was recently proposed by Brock and colleagues [18], where they show that only Asp49 and Arg126 are catalytically critical. Moreover, Hammarberg *et al*., [19] investigated the role of Arg126 in MPGES1, where they observed that the mutation of Arg to Ala transforms MPGES1 catalytic activity to a prostaglandin F_2α_ (PGF_2α_) synthase, which was also confirmed by Brock *et al*., [18]. The enzyme apparently is altered from an isomerase to a reductase.

Based on the above mentioned structural and mechanistic data we set out to investigate the catalytic roles of Asp49, Arg73, Arg126 and Ser127, all of which are near to, or coordinating the GSH molecule. We generated six variants in an *E*. *coli* expression system; Asp49Ala, Arg73Ala, Arg73Leu, Arg126Ala, Arg126Leu and Ser127Ala. Membrane fractions were prepared for activity assessment by the rapid malondialdehyde– 2-thiobarbituric acid (MDA-TBA) activity assay [20]. In addition, liquid chromatography coupled to mass spectrometry (LC-MS) was applied for prostanoid profiling.

## 2. Material and Methods

### 2.1. Site-directed mutagenesis

Residues coordinating the GSH molecule; Asp49, Arg73, Arg126, and Ser127 were chosen for mutagenesis. The expression vector pSP19T7LT harboring the coding sequence for human MPGES1 [6] was subjected to site-directed mutagenesis using the GeneTailor^™^ system from Invitrogen (Stockholm, Sweden). The methylated plasmid DNA was amplified by polymerase chain reaction with two overlapping specific mutation primers. Plasmid DNA with the correct mutations, i.e. Asp49Ala, forward: 5' AAGGCCTTTGCCAACCCCGAG**GCG**GCCCTGAGACACGGAGGCCCC-3'; Arg73Ala, forward: 5'-GAACGCTGCCTCAGGGCCCAC**GCG**AACGACATGGAGACCATCTAC-3'; Arg73Leu, forward: 5'-GAACGCTGCCTCAGGGCCCAC**GAC**AACGACATGGAGACCATCTAC-3'; Arg126Ala, forward: 5'-AAGCTGCGGGCACCCATC**CGC**GCGGTGACCTACACCCTGGCCCAG-3'; Arg126Leu, forward: 5'-GGGAAGCTGCGGGCACCCATC**GCG**TCCGTGACCTACACCCTGGCC-3'; Ser127Ala, forward: 5'-GGGAAGCTGCGGGCACCCATCGAC**TCC**GTGACCTACACCCTGGCC-3'; were thereafter transformed into the *E*. *coli* DH5α-T1R-competent cells. DNA sequencing was used to confirm and verify the mutations and the insert sequence.

### 2.2. Protein expression in *E*. *Coli* and subcellular fractionation

The different constructs were all transformed into the *E*. *coli* BL21Star^™^ (DE) pLysS-competent cells. A single bacterial colony was used to inoculate with 5ml LB medium containing 100μg/ml ampicillin and 20μg/ml chloramphenicol and incubated overnight at 37°C with 300rpm shaking. Overnight cultures were diluted 1:50 in 200ml pre-warmed Terrific Broth (TB) medium containing the same concentration of antibiotics. Cultures were let to grow at 37°C with 300rpm shaking until OD600 reached 0.45–0.6 followed by induction with 1mM isopropyl β-D-thiogalactopyranoside for 3h. Cells were harvested by centrifugation at 4,600rpm for 15minutes at 4°C. Cell pellets from 50ml culture were resuspended in 1ml TSEG buffer (15mM Tris-HCl pH 8.0, 0.25M Sucrose, 0.1mM Ethylenediaminetetraacetic Acid (EDTA), 1mM GSH) on ice. Lysis of the cells was carried out by freeze-thawing 5 times with liquid nitrogen and a 37°C water bath. 10mM MgCl_2_ and 0.4μg/ml DNAse was added and left to incubate on ice for 30 minutes to hydrolyze DNA; followed by six 30 second ultrasonic sonication pulses at 60% power. Cell debris was removed by centrifugation at 1,000rpm for 15 minutes at 4°C. Supernatants were ultracentrifuged at 35,000rpm for one hour at 4°C. Membrane pellets were resuspended in 1ml resuspension buffer (20mM Sodium Phosphate buffer pH 8.0, 15mM NaCl, 10% glycerol, 1mM GSH). The total protein concentration was measured by the Bradford technique at 620nm [21]. As standards, bovine serum albumin (BSA) was used at different concentration yielding at 0.0625, 0.125, 0.25, 0.5 and 1μg/μl.

### 2.3. Malondialdehyde– 2-thiobarbituric acid assay (MDA-TBA) for MPGES1 activity, measured in the membrane fraction of *E*. *Coli*

PGH_2_ converting activity of MPGES1 was determined as previously described [20]. Briefly recombinant, membrane bound, wildtype (WT) or MPGES1 variants was incubated for 60 seconds at room temperature (RT) in the presence of 10μM PGH_2_. The reaction was carried out in 0.1M sodium phosphate buffer, pH 8.0, supplied with 2.5mM glutathione (GSH) on wet ice. Instant reaction stop was achieved by substrate depletion after the incubation, by the addition of 100μl stop solution (containing 25mM FeCl_2_ and 50mM citric acid, pH 3.0). Unmetabolized PGH_2_ was converted to 12-(S)-hydroxy-8, 10-*trans*-5-*cis*-heptadecatrienoic acid (12-HHT) and malondialdehyde (MDA). Thiobarbituric acid (TBA) solution (containing 128.4mM TBA and 148.15mM citric acid) was subsequently added and the reaction mixture was incubated at 80°C over 30 minutes to allow formation of the fluorescent MDA-TBA conjugate. Conjugate concentration was analyzed by fluorescence measurements using a Victor3V 1420 Multilabel counter (Perkin Elmer, Waltham, Massachusetts, USA) at excitation 485 nm and emission 545 nm.

### 2.4. Microsomal prostaglandin E_2_ synthase 1 activity assay, measured in the membrane fraction of *E*. *Coli*

Appropriate dilutions of total membrane fraction protein in duplicates were made in activity assay buffer containing 0.1M potassium phosphate buffer pH 7.5 (0.1% Triton X-100, 1% glycerol and 2.5mM GSH) to obtain a final concentration of 300μg protein/ml. These were incubated with PGH_2_ (final concentration 10μM), GSH (2.5mM final concentration) for 60 seconds at room temperature. After incubation, the reaction was immediately stopped by adding 400μl of stop solution containing 25mM FeCl_2_ and 50mM citric acid. The remaining PGH_2_ is converted mainly to 12‐ HHT and MDA by the ferric ions. A deuterated internal standard of PGE_2_, PGD_2_, PGF2_α_, TXB_2_, 6-keto-PGF_1α_, 13,14-dihydro-15-keto-PGE_2_ and 15-deoxy-Δ 12, 14 PGJ_2_ (Cayman Chemicals, Michigan, USA) was added to each sample. The WT and variant enzyme *E*. *coli* membrane fractions were analyzed. Denatured (boiled for 10 minutes) samples were incorporated as negative controls.

### 2.5. Liquid chromatography coupled to mass spectrometry (LC-MS) analysis of the *E*. *Coli* membrane incubations

Solid phase extraction was applied to each sample of the PGE_2_ activity assay described above. 30mg Oasis HLB single extraction columns (Waters Corporation, Milford, MA, USA) were pre-conditioned with 1ml methanol and then 1ml 0.05% formic acid in MilliQ water. 600μl of samples were added to the columns and washed with 1ml 5% methanol acidified with 0.05% formic acid in MilliQ water and subsequently eluted into 1ml methanol followed by evaporation under vacuum. Samples were re-dissolved in 50μl 7% acetonitrile in MilliQ water prior to analysis by using a Waters 2795 HPLC (Waters Corporation, MA, USA) coupled to a triple quadrupole mass spectrometer (Acquity TQ Detector, Water Corporation). 40μl aliquots were injected to a Synergi Hydro-RP column (100mm x 2mm i.d, 100Å pore size and 2.5μm particle size, Phenomenex, CA, USA) applying a 45 minute stepwise linear gradient. MilliQ water was used as mobile phase A and acetonitrile, 0.05% formic acid as mobile phase B. In the first step with the duration of 9 minutes’ mobile phase B was increased from 10% to 25%, then to 45% during 22 minutes in step two, and furthermore to 70% during 5 minutes in step three. A washing step of 90% mobile phase was applied and then re-equilibrated at 10% mobile phase. Detection of analytes was done in multiple reaction monitoring (MRM) mode followed by analysis with MassLynx software, version 4.1, using internal standard calibration for quantification of PGE_2_, PGD_2_, PGF_2α_, TXB2, 6-keto-PGF_1α_.

### 2.6. Western Blot analysis of *E*. *Coli* membrane fractions

Bacterial pellets were lysed in lysis buffer (containing 50mM Tris-HCl pH 7.4, 100mM KCl, 1mM EDTA, 1mM DTT, 0.1% Igepal) supplemented with 1x complete protease inhibitor cocktail (Roche Diagnostics GmbH, Mannheim, Germany) on ice for 30 minutes. Determination of total protein concentration was carried out with the NanoDrop technique (NanoDrop 2000c; Thermo Scientific, Stockholm, Sweden). Equal amounts of protein were separated by gel electrophoresis using an NuPage^®^ Novex^®^ Bis-Tris gel system (Invitrogen AB, Lidingo, Sweden). Proteins were transferred to a polyvinylidene difluoride (PVDF) membrane by using a Trans-Blot SD semi-dry transfer cell (Bio-Rad Laboratories AB, Uppsala, Sweden). Membrane was blocked with 5% milk (Bio-Rad Laboratories AB, Uppsala, Sweden) in phosphate-buffered saline (PBS) (0.1% Tween 20) for 30 minutes at RT on a rocker. Blocking of membrane was followed by overnight incubation of primary antibody at 4°C (1:250; polyclonal MPGES1; Cayman Chemicals, MI, USA) and secondary antibody for one hour at RT (HRP-coupled anti-rabbit IgG from donkey; GE Healthcare, Stockholm, Sweden). After incubation of each antibody the membrane was washed three times for 10 minutes each time in PBS (0.1% Tween 20). Bands were visualized by ECL kit (GE Healthcare, Stockholm, Sweden) on film (Amersham Hybond; GE Healthcare, Buckinghamshire, UK). MPGES1 purified according to Thorén *et al*., [22] was used at different concentrations as standards, yielding at 75, 100, 150, 200 and 450ng. Densitometry measurements on the resulting WB film were performed using the program ImageJ and a non-linear regression curve was fit to the standards.

### 2.7. Protein expression in *Baculovirus* infected *SF9 cells* and protein purification

The Ser127 residue was mutated to an Ala or Cys residue by using the QuikChange II XL site-directed mutagenesis Kit (Agilent Technologies, Santa Clara, California, USA) according to the manufacturer’s instructions. The mutated protein was expressed in XL1 blue supercompetent cells and plasmid DNA was isolated and purified using a QIAprep Spin Miniprep Kit (Qiagen, Hilden, Germany). The mutation was confirmed by sequencing (GenHunter, Nashville, TN).

The human MPGES1 cDNA containing a C-terminal His_6_ tag was subcloned into a pVL 1392 transfer vector. Incorporation of the cDNA was confirmed with sequencing (GenHunter, Nashville, TN). Approximately 2μg of the vector was co-transfected with BestBac linearized *Baculovirus* DNA (Expressions Systems, Davis, CA). The *Baculovirus* containing the MPGES1 cDNA was amplified and then used to infect *Spodoptera frugiperda* insect (SF9) cells. Large suspension cultures were infected with virus stock at a cell density of 1.2 x 10^6^ cells/ml and incubated at 27°C with shaking at 140rpm. Cell cultures were harvested 72 hours post-infection by centrifugation at 4,000rpm for 20 minutes. For every gram cell pellet 10ml of lysis buffer (containing 15mM Tris-HCl, pH 8.0, 250mM Sucrose, 1mM GSH, 0.1mM EDTA, 10% Glycerol) was added. Cells were stirred at 4°C for approximately two hours and then sonicated for 4 x 30 seconds, 50% duty cycle. Cell lysate was cleared by centrifugation at 10,000rpm, 4°C for one hour. The supernatant was then centrifuged at 35,000rpm, 4°C for 1.5 hours to isolate the microsomal fraction, which was then resuspended in extraction buffer (containing 50mM KH_2_PO_4_, pH 8.0, 300mM KCl, 1mM GSH, 5mM imidazole, 0.5% β-DDM, 10% Glycerol, and 1 x protease inhibitor cocktail tablet). The membrane fraction was stirred overnight at 4°C for complete solubilization. Insoluble material was cleared by an additional centrifugation at 35,000rpm, 4°C for one hour. The solubilized membrane fraction was gently stirred with Ni-NTA resin, equilibrated with extraction buffer, at 4°C for one hour and added to a gravity column. Proteins in the lysate that could bind non-specifically to the Ni-NTA resin, were washed from the resin with a buffer containing 50mM KH_2_PO_4_, pH 8.0, 300mM KCl, 1mM GSH, 60mM imidazole, 0.05% β-DDM, 10% Glycerol. MPGES1 was then eluted from the resin with the same buffer at pH 7.0, containing 250mM imidazole. The elution fraction was concentrated using centrifugal concentrator tubes with a molecular weight cutoff of 50kDa and then dialyzed overnight at 4°C in a buffer containing 25mM KH_2_PO_4_, pH 7.0, 1mM GSH, 0.05% β-DDM, 10% glycerol. The enzyme was then added to a gravity column containing SP Sepharose resin equilibrated with the aforementioned buffer. The column was washed with 10 volumes of the same buffer. MPGES1 was then eluted with the same buffer also containing 100mM KCl. The elution fraction was concentrated and then injected onto a Sephacryl gel filtration column. 120ml of buffer (50mM KH_2_PO_4_, pH 7.0, 150mM KCl, 1mM GSH, 0.05% β-DDM, 5% glycerol) was used to elute the enzyme. Fractions containing MPGES1 were collected and concentrated to approximately 60μM. The enzyme was dialyzed overnight at 4°C in a final buffer containing 50mM KH_2_PO_4_, pH 7.0, 200mM KCl, 1mM GSH, 0.05% β-DDM, 10% glycerol. The purity of the enzyme was assessed using SDS-PAGE. Furthermore, a monodisperse peak from gel filtration chromatography (data not shown) indicated that the enzyme was purified in its trimeric state and homogeneously folded. Pure MPGES1 was aliquoted into 1.5 ml tubes and stored at -20°C for experiments.

### 2.8. Glutathione transferase (GST) activity assay of purified MPGES1 expressed in *Baculovirus* infected *SF9 cells*

The enzymatic activity of purified MPGES1 can be measured also by the conjugation of GSH to the electrophilic substrate 1-chloro-2,4-dinitrobenzene (CDNB) [22]. This enzymatic activity was measured spectroscopically at 340 nm and 22°C using a Beckman Coulter DU 800 Spectrophotometer. Each sample had a total reaction volume of 500l and contained 100mM phosphate buffer, pH 6.5, 5mM GSH, 2mM CDNB, 0.1% β-DDM and either 1 or 5μM of purified enzyme. Each sample contained 1% acetonitrile, the solvent for CDNB. The spontaneous conjugation of CDNB to GSH was subtracted from each enzymatic reaction and the specific activity values were calculated based on the slope of the initial linear portion of the absorbance curve.

### 2.9. Enzyme activity assay for purified WT and Ser127 MPGES1 variants expressed in *Baculovirus* infected *SF9 cells*

Isomerization of PGH_2_ to PGE_2_ was measured by utilizing LC-MS/MS as previously described [23,24]. PGH_2_ (Cayman Chemical, Ann Arbor, MI, USA) was distributed into 10μl aliquots from a 284μM stock in eppendorf tubes kept on dry ice. For full-stopped reactions, PGH_2_ aliquots were equilibrated at RT for approximately 30 seconds and the reaction was stopped by adding 100μl of stop buffer containing 100mM FeCl_2_, 500mM KH_2_PO_4_, pH 2.0 and was returned to wet ice. For spontaneous reactions, PGH_2_ aliquots were equilibrated on wet ice for approximately 30 seconds and 100μl reaction buffer (100mM KH_2_PO_4_, pH 7.4, 1mM GSH, 0.05% β-DDM, 1% Glycerol) was added with no enzyme. The mixture was vortexed and the reaction proceeded at RT for one minute. The reaction was stopped by adding stop buffer and was returned to wet ice. For the enzymatic reactions, the same procedure was followed, except with the addition of 100nM enzyme into the reaction buffer. The GSO^-^_3_ preparation was similar to the GSH preparation. Once the cells were lysed and the membrane fraction was isolated, the membranes were solubilized in a buffer containing 1mM GSO^-^_3_. Thereafter, the enzyme was purified with all buffers containing 1mM GSO^-^_3_. After each reaction was stopped, the products were isolated with solid-phase extraction using Oasis HLB Extraction Cartridges (Waters Corporation, Milford, MA, USA). The products were washed with 5% methanol and eluted with 1ml of 100% methanol. The methanol was evaporated using N_2_ gas from the prostanoid products and they were reconstituted in 20% CH_3_CN in H_2_O. Each sample was analyzed using reversed-phase LC-MS/MS with a Phenomenex Luna 3.0μm, C18 (2), 100Å column (150 x 2.00mm) equipped with a Phenomenex Security Guard Cartridge Kit, eluting at a flow rate of 350μl/min using mobile phase A (H_2_O + 0.1% formic acid) and mobile phase B (CH_3_CN + 0.1% formic acid). A gradient of 31.2 to 42.5% B was running over 14.3 minutes. Using negative electrospray ionization, the detection of PGE_2_ was confirmed by single-reaction monitoring (SRM) of the transition *m/z* 351.2 to 271.2 on a ThermoFinnigan TSQ triple-quadrupole mass spectrometer. The amount of PGE_2_ was quantified from integration of its peak area relative to the internal standard, 11β-PGE_2_ (Cayman Chemical, MI, USA). The percentage activity of variants was normalized to the WT MPGES1 activity set at 100%.

## 3. Results and Discussion

### Choice of mutation targets

Based on results and predictions from previous structural studies [17–19], we chose to investigate the effect of substituting amino acid residues Asp49, Arg73, Arg126 and Ser127 on MPGES1 catalytic activity. Previous mutagenesis studies on MPGES1 focused on residues close to the location of the GSH binding site in the protein and were largely uninformative regarding mechanism [25,26], the exceptions being the identification of the obligatory catalytic role of Arg126 (19) and residues that determine species differences between rodents and humans for inhibitor binding [27]. Especially the latter have practical consequences for pharmacological drug development, limiting the utility of several rodent model systems for inflammatory disease (9). The more recent high-resolution structure determined by Sjögren *et al*., [17] allowed for a more precise hypothesis to be taken on mechanistically important residues. The authors suggested that Arg126 and Asp49 interact and are involved in proton abstraction from PGH_2_ whereas Ser127 was suggested to lower the pKa of the GSH thiol promoting thiolate stabilization. They also describe that the side chain of Arg73 blocks the connection between the central cavity and the active site of MPGES1. Further, both Hammarberg *et al*., [19] and Brock *et al*., [18] observed that the mutation of Arg126 to Ala transforms MPGES1 catalytic activity into PGF_2α_ synthase. Based on the accumulated findings described above, we aimed to explore the amino acids critical to the catalytic mechanism of MPGES1 through site-directed mutagenesis and activity measurements.

### Activity of WT and variant MPGES1

**Fig 1:**
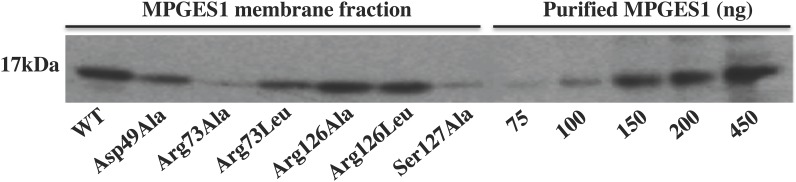
Western Blot analysis of WT MPGES1 and variants Asp49Ala, Arg73Ala, Arg73Leu, Arg126Ala, Arg126Leu, Ser127Ala, expressed in *E*. *Coli*. 40μg of membrane fraction was loaded into each well. The exposure time was 5 minutes. As a positive control purified MPGES1 was loaded at different concentrations, yielding: 75ng, 100ng, 150ng, 200ng and 450ng.

**Fig 2:**
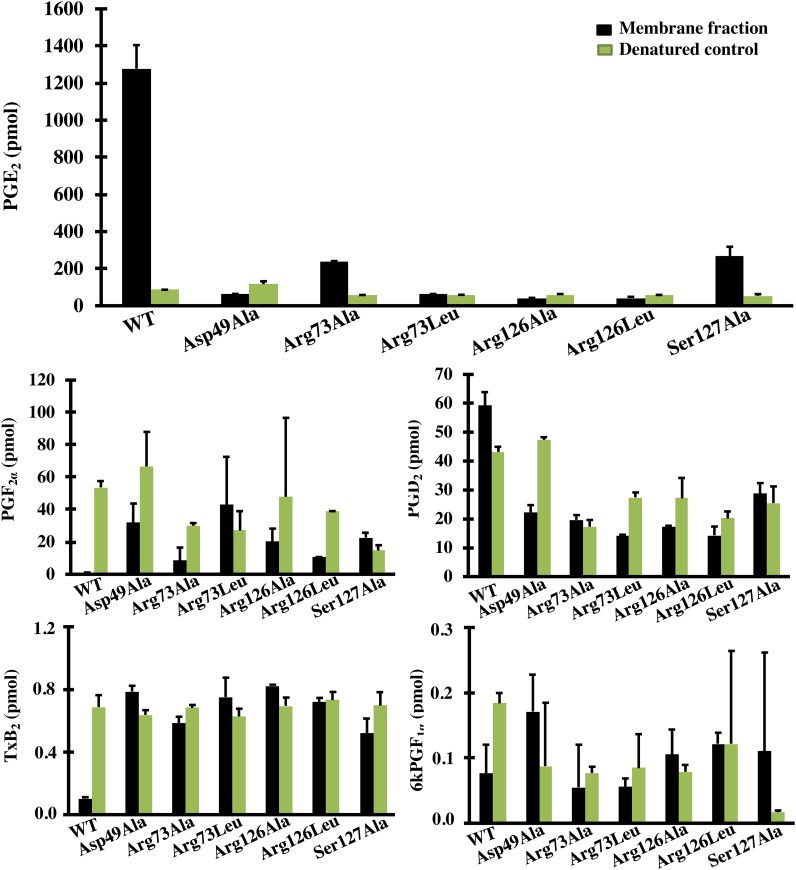
Prostanoid profiles for WT MPGES1 and variants Asp49Ala, Arg73Ala, Arg73Leu, Arg126Ala, Arg126Leu, Ser127Ala, expressed in *E*. *Coli*. Prostanoid production was measured by LC-MS/MS after 60 seconds incubations of membrane fractions with PGH_2_ (10μM final concentration) and GSH (2.5mM final concentration) at room temperature. Denatured enzymes by boiling were incorporated in the activity assay as controls. All prostanoid measurements of membrane fractions are expressed as mean ± SD from two independent experiment performed in duplicates.

The WT and variant forms of MPGES1 were first expressed in the *E*. *coli* expression system and the amounts of expressed protein were quantified by Western Blot analysis (Fig 1). All constructs expressed MPGES1 albeit to varying degrees. Enzymatic activities of the WT MPGES1 and variants were first screened by a simple activity assay measuring remaining substrate (described in section 2.4 of Materials and Methods, data not shown), followed by detailed measurement employing prostanoid profiling by LC-MS (Fig 2). Only WT MPGES1, Arg73 and Ser127 variants catalyzed the formation of PGE_2_ to significantly higher amounts than their negative controls (denatured protein, boiled for 10 minutes). Neither WT MPGES1 nor the variants altered the background level amounts of the other prostanoids quantified by LC-MS (Fig 2). The specific activity levels (Table 1) that we measured in our *E*. *coli* system are in range with those observed by Pawelzic *et al*., [27], but significantly lower than the specific activity initially observed by Thorén *et al*., (7) with purified MPGES1.

**Table 1:** Specific activity of WT MPGES1 and variants Asp49Ala, Arg73Ala, Arg73Leu, Arg126Ala, Arg126Leu and Ser127Ala, expressed and measured in *E*. *Coli* membrane fractions.

Variants	Specific PGE_2_ activity (μmol min^−1^ mg^−1^)	Retained activity (% of WT activity)
Asp49Ala	0	0
Arg73Ala	0.32	50
Arg73Leu	0	0
Arg126Ala	0	0
Arg126Leu	0	0
Ser127Ala	0.40	63
WT	0.63	

### The role of Ser127

**Fig 3:**
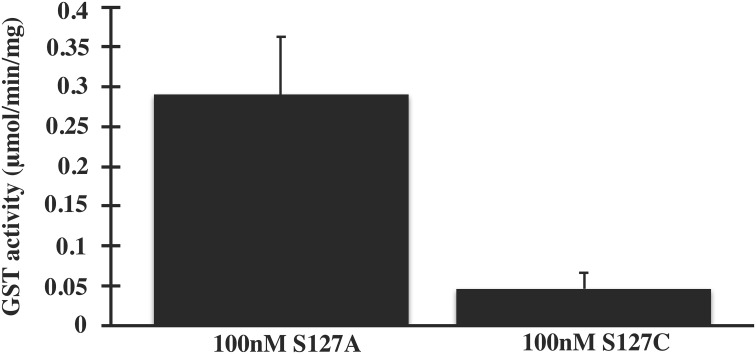
Glutathione transferase (GST) activity assay of purified MPGES1, expressed in *Baculovirus* infected *SF9 cells*. The enzymatic activity of purified MPGES1 was measured by the conjugation of GSH to the substrate 1-chloro-2,4-dinitrobenzene (CDNB). The spontaneous conjugation of CDNB to GSH was subtracted from each enzymatic reaction and the specific activity values were calculated based on the slope of the initial linear portion of the absorbance curve. The GST activity is expressed as mean ± SD from two independent experiment performed in triplicates.

**Fig 4:**
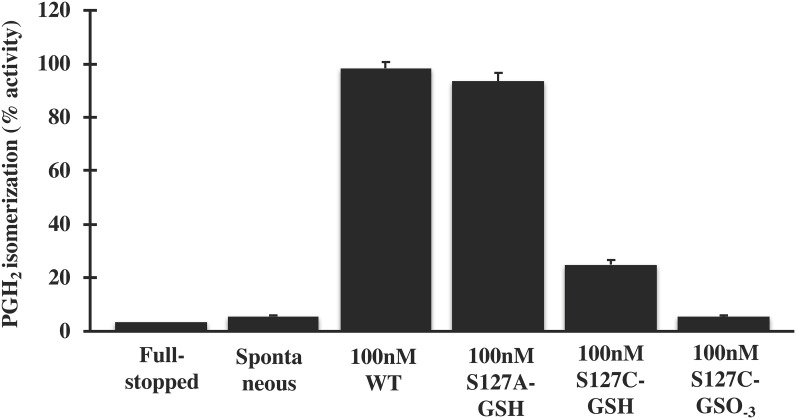
Enzyme activity assay for purified WT MPGES1 and Ser127 variants, expressed in *Baculovirus* infected *SF9 cells*. The isomerization of PGH_2_ to PGE_2_ was verified with detection of PGE_2_ by LC-MS/MS after 60 seconds incubation of 100nM enzyme with PGH_2_ (25.8μM final concentration) and GSH (1mM final concentration) at room temperature. The PGH_2_ isomerization activity is expressed as mean ± SD from two independent experiment performed in triplicates.

Structural data [17] convincingly suggested that Ser127 might be an important residue for the catalytic activity of MPGES1. Our results however, show that mutation of Ser127 to Ala only lowers the catalytic activity of MPGES1 measured in *E*. *coli* membranes (Table 1, Figs 1 and 2). As the expression system might influence the behavior of the enzyme, we decided to investigate by applying a second expression system, Baculovirus expression system. In addition, we also analyzed the behavior of Ser127Cys variant. The activity of purified WT MPGES1 and Ser127 variant enzymes from SF9 cells was measured both by a GST assay (Fig 3) and was quantified by PGH_2_ isomerization (Fig 4). The Ser127Ala variant retained full activity in its purified form, regardless of assay employed (Figs 3 and 4). We therefore conclude that the Ser127 residue is not necessary for catalytic activity, confirming the data of Brock *et al*., [18]. The alanine residue, being small, does not interfere with critical steps in the catalytic cycle. However, the Ser127Cys variant displayed considerably lowered activity (20% of WT MPGES1) based on GST activity and PGH_2_ isomerization assay (Figs 3 and 4). The lower activity is consistent with close proximity to the active site and steric hindrance emanating from the larger cysteine electron cloud. The cysteine thiol of Ser127Cys could in principle replace the GSH thiol and support catalysis, admittedly a radical suggestion. Purification of the Ser127Cys variant in the presence of glutathione sulfonate, to deplete the enzyme completely of GSH, yielded inactive enzyme (Fig 4). Therefore, we conclude that GSH as a cofactor is absolutely required for the enzymatic activity of MPGES1.

In conclusion, Ser127Ala variant activity is obtained regardless of expression system, in fact the activity of WT MPGES1 and variants are equal in the purified enzyme variants (Figs 3 and 4). The activity is somewhat lower of the variant compared to WT MPGES1 when measured in *E*. *coli* membranes (Table 1). As post-translational modifications have not been detected in MPGES1, the membrane lipid composition might be accounting for the difference of purified enzyme, but it should be kept in mind that quantitation of protein levels from Western blots is not entirely precise.

### The roles of Asp49 and Arg126

We confirm that mutation of Asp49 to alanine and Arg126 to either alanine or leucine resulted in complete loss of activity (Table 1, Fig 2) confirming a central role in catalysis for these residues. Our data confirm those of Brock *et al*., [18]. However, in contrast to the observations of Hammarberg *et al*., [19] and Brock *et al*., [18] we could not detect any PGF_2α_ formation when examining the prostanoid profile by LC-MS (Fig 2), even when the reaction with the Arg126Ala variant was left to proceed for 10 minutes, no PGF_2α_ formation was detected (data not shown). The reason for the discrepancy is not known but could be due to that different expression systems were used in the experiments (*E*. *coli* vs SF9 cells).

**Fig 5:**
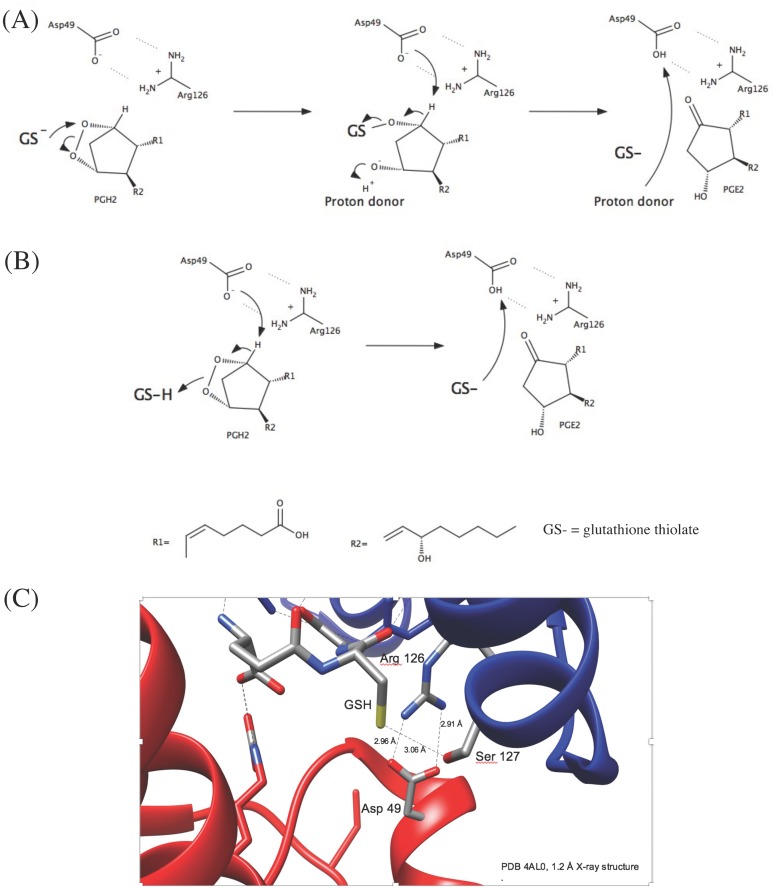
Suggested chemical mechanism of PGH_2_ isomerization to PGE_2_ by MPGES1 and its active site structure highlighting the amino acids altered. (A) The thiolate of glutathione (GSH) could be stabilized by Arg126 and attack the C9 oxygen of the PGH_2_ endoperoxide forming a sulfenic acid ester. An unidentified proton donor protonates the developing C11 oxyanion. This is followed by proton abstraction at C9 via Asp49. A carbonyl forms and the oxygen sulfur bond is broken forming PGE_2_. The leaving GSH thiolate could again be stabilized by Arg126. The unidentified proton donor could then take up the proton from Asp49. (B) The reaction starts by proton abstraction at C9 via Asp49. A carbonyl forms and the endoperoxide bridge is broken. The thiol of GSH functions as a proton donor to the developing C11 oxyanion. After that the proton taken up by Asp49 can reprotonate the GSH thiolate. (C) The interaction between MPGES1 and GSH highlighting the positions of Asp49, Arg126 as well as Ser127 that was proposed to stabilize the GSH thiolate.

We propose two alternative chemical reaction mechanisms for the MPGES1 catalyzed PGE_2_ synthesis (Fig 5A and 5B). The first one involves an attack of the GSH thiolate (in a glutathione peroxidase like mechanism) on the C9 oxygen in the endoperoxide bridge forming a sulfenic acid ester. The developing oxyanion at C11 receives a proton (no donor specified). Then follows proton abstraction at C9 by Asp49 where a carbonyl forms as the oxygen sulfur bond is broken. Arg126 could stabilize the leaving GSH thiolate and the reactions result in the formation of PGE_2_. The second alternative is a concerted mechanism where Asp49 initiates the reaction by proton abstraction at C9, formation of the carbonyl and simultaneous splitting of the endoperoxide with the protonated GSH donating a proton to the developing oxyanion resulting in the formation of PGE_2_ (Fig 5B). Subsequently GSH could be deprotonated from Asp49.

### A role for a GSH thiolate

Although the structural data by Sjögren *et al*., [17] suggested a role for Ser127 in the stabilization of a GSH thiolate, our mutagenesis data opens for other alternatives regarding chemical mechanism and Arg126 is now a stronger candidate for this function (Fig 5C). However, so far there is no experimental data that directly demonstrates stabilization of a GSH thiolate in the active site of MPGES1. Unless this can be demonstrated, as has been done with other members of the MAPEG family [28–30], a different chemical mechanism, which could even involve the GSH thiol as a proton donor (Fig 5B), cannot be excluded.

One could argue that, as MPGES1 catalyzes the conjugation of GSH to CDNB, the enzyme must stabilize a GSH thiolate to be able to perform this chemistry. This is certainly a valid argument, however, even if the pK_a_ of GSH is not lowered in the active site, a small fraction of the GSH will always be in the thiolate form (approximately 2% at physiological pH). Thus the CDNB reaction can be maintained by this fraction as the GSH thiolate rapidly re-equilibrates. This scenario is actually reminiscent of the behavior of the closest MAPEG relative MGST1. For this enzyme, in the steady state reaction with CDNB, the GSH thiolate is so rapidly reacting (and slowly re-forming) that it is a minor intermediate during catalysis. In other words, a small fraction of GSH thiolate in MPGES1 could arguably account for catalysis with CDNB. In conclusion, the residue (Ser127) that from a structural point of view is the best candidate for a defined catalytic role has now been ruled out, whereas the Arg residue, that performs this function in other MAPEG members, now is a more attractive candidate for GSH thiolate stabilization. Until the presence of a GSH thiolate has been demonstrated however, this conjecture is purely hypothetical.

### Amino acid residues in the GSH binding site

Sjögren *et al*., [17] identify several other residues in proximity to GSH. Here we show that Arg73 most likely does not have a catalytic role (Table 1, Fig 2) as the Arg73 variant retains most of the catalytic activity. A structural role is indicated as the alanine variant retains partial activity whereas the leucine variant does not. Tyr117 mutated to Ala had 1% activity wheras the phenylalanine variant was fully active, indicating a structural role also for this residue [31]. We previously demonstrated that Glu77 is essential for activity. Others have shown that mutation of Tyr130, suggested to bind to GSH via pi stacking interactions, results in lowering of activity (to approximately 15%) [26]. In general, it appears that residues contacting GSH can be altered with varying effects on activity. Our characterization of absolutely essential and non-essential residues must now be combined with direct observation of the state of GSH thiol ionization. Such studies will aid in determining the chemical mechanism of MPGES1 for which there are several different proposals [17,18,31] (Fig 5A and 5B).

In conclusion, our findings confirm the hypothesis that Arg126 and Asp49 are essential for the catalytic mechanism of MPGES1. Furthermore, we conclude that residues Arg73 and Ser127 are not catalytically important for MPGES1. Finally, in our expression system the Arg126Ala variant does not catalyze the conversion of PGH_2_ into PGF_2α_. Our findings provide information on important residues for the catalytic activity of MPGES1. However, further experiments are needed to uncover the chemical mechanism of this enzyme.
